# Endoscopic management of sinonasal tumours in the Nordic university hospitals: a survey

**DOI:** 10.1007/s00405-023-08229-w

**Published:** 2023-09-21

**Authors:** Carl Korsström, Markus Lilja, Lalle Hammarstedt-Nordenvall, Antti Mäkitie, Aaro Haapaniemi

**Affiliations:** 1 Department of Otorhinolaryngology-Head and Neck Surgery, University of Helsinki and HUS Helsinki University Hospital, P.O. Box 263, 00029 HUS Helsinki, Finland; 2 Division of Ear, Nose and Throat Diseases, Department of Clinical Sciences, Intervention and Technology, Karolinska Institutet and Karolinska University Hospital, Stockholm, Sweden; 3 Department of Head-, Neck-, Lung- and Skin Cancer, Theme Cancer, Karolinska University Hospital, 17164 Stockholm, Sweden; 4 Research Program in Systems Oncology, Faculty of Medicine, University of Helsinki, Helsinki, Finland

**Keywords:** Endoscopy, Practice guidelines as topic, Nose neoplasms, Paranasal sinus neoplasms, Paranasal sinuses, Scandinavian and Nordic countries

## Abstract

**Purpose:**

The Nordic countries (27 M) all have comparable, publicly funded healthcare systems, and the management of sinonasal tumours is centralised to the 21 university hospitals. We sought to assess and compare the treatment practice of sinonasal tumours across the Nordic countries.

**Methods:**

A web-based questionnaire was sent to all university hospital departments of otorhinolaryngology—head and neck surgery in the Nordic countries.

**Results:**

Answers were obtained from all 21 Nordic university hospitals. The endoscopic approach was widely utilised by all, with most (62%) centres reporting 3–4 surgeons performing endoscopic sinonasal tumour surgery. Finland reported the lowest rates of centralisation among university hospitals despite having the highest number of 0.1–1 M catchment population hospitals. Most centres (88%) opted for the endoscopic approach in a patient case warranting medial maxillectomy. In a case of a Kadish C esthesioneuroblastoma, most (52%) of the centres preferred an endoscopic approach. Most centres (62%) reported favouring the endoscopic approach in a case describing a juvenile angiofibroma. Regarding a case describing a sinonasal undifferentiated carcinoma, consensus was tied (38% vs. 38%) between endoscopic resection followed by postoperative (chemo)radiotherapy (RT/CRT) and induction chemotherapy followed by RT/CRT or surgery followed by RT/CRT.

**Conclusion:**

Endoscopic approach was widely utilised in the Nordic countries. The case-based replies showed differences in treatment practice, both internationally and nationally. The rate of centralisation among university hospitals remains relatively low, despite the rarity of these tumours.

**Supplementary Information:**

The online version contains supplementary material available at 10.1007/s00405-023-08229-w.

## Introduction

Sinonasal tumours are rare with a reported annual incidence of less than 1/100,000 and sinonasal malignancies constituting under 1% of all malignancies [1–4]. The rareness and heterogeneity of this disease entity complicate the collection of large uniform cohorts, and therefore, generally accepted treatment guidelines are lacking.

Endoscopic surgery of sinonasal tumours was first introduced as an alternative to traditional, open surgery in the 1990s [5] and was deemed viable practice by the European Rhinological Society in a position paper in 2010 [2]. The rising popularity of endoscopic resection is due to the reduced invasiveness, and most clinical outcome seem comparable to open surgery [6]. Some possible benefits regarding complication rate and postoperative length of stay have been reported [2, 7, 8].

The Nordic countries (27 M), i.e. Denmark, Finland, Iceland, Norway, and Sweden all have rather uniform, publicly funded healthcare systems. The management of sinonasal malignancies is centralised to the 21 university hospitals and national management guidelines are utilised.

We conducted a survey in all the five Nordic countries, with the primary aim to assess treatment practice of sinonasal tumours, with a focus on the use of endoscopic approach. Our secondary aim was to assess the degree of national centralisation of the treatment of sinonasal tumours among university hospitals.

## Materials and methods

A web-based questionnaire (see supplementary information) was sent out to all 21 Nordic university hospitals, departments of otorhinolaryngology—head and neck surgery (ORL-HNS): Denmark (Aalborg, Aarhus, Copenhagen, Odense), Finland (Helsinki, Kuopio, Oulu, Tampere, Turku), Iceland (Reykjavik), Norway (Bergen, Oslo, Tromsø, Trondheim), and Sweden (Gothenburg, Linköping, Lund, Stockholm, Umeå, Uppsala, Örebro).

The otorhinolaryngologist—head and neck surgeons responsible for the management of sinonasal tumours at each centre were asked to fill in the questionnaire. The questionnaire remained open from April 2022 to November 2022 and reminders were sent to the non-respondent centres to secure the completeness of data.

Data recorded through the survey consisted of general information on the centre: referral area, number of surgeons performing endoscopic surgery of sinonasal tumours, availability of image-guided navigation, and speciality of the responder. Care pathway of choice for selected tumours (juvenile angiofibroma [JNA], inverted papilloma [IP], malignant sinonasal tumours, and sinonasal sarcoma) was recorded. Recorded data regarding endoscopic surgery included routine application of endoscopic approach for sinonasal tumours, whether endoscopic surgery of sinonasal tumours is centralised to certain surgeons, which speciality performs transsphenoidal surgery, and whether IPs are managed mainly endoscopically. Lastly, the respondent was asked to report the preferred course of action in a series of four fictional case-based questions. Distance and travel time by car to the nearest neighbouring university hospital within the same country was calculated using Google Maps (Google).

## Results

### General information

Responses from all 21 (100%) university hospitals in the five Nordic countries were obtained in November 2022. All respondents reported ORL-HNS as their speciality. Out of the Nordic university hospitals, 4 (19%) have a referral area of 100,000–500,000 inhabitants, 7 (33%) encompass 500,001–1,000,000 inhabitants, 6 (29%) encompass 1,000,001–2,000,000 inhabitants, and 4 (19%) encompass more than 2,000,000 inhabitants. Seven of the centres reported 1–2 surgeons performing endoscopic sinonasal tumour surgery, 13 (62%) reported 3–4 and 1 (5%) more than 5 surgeons, respectively.

**Fig. 1:**
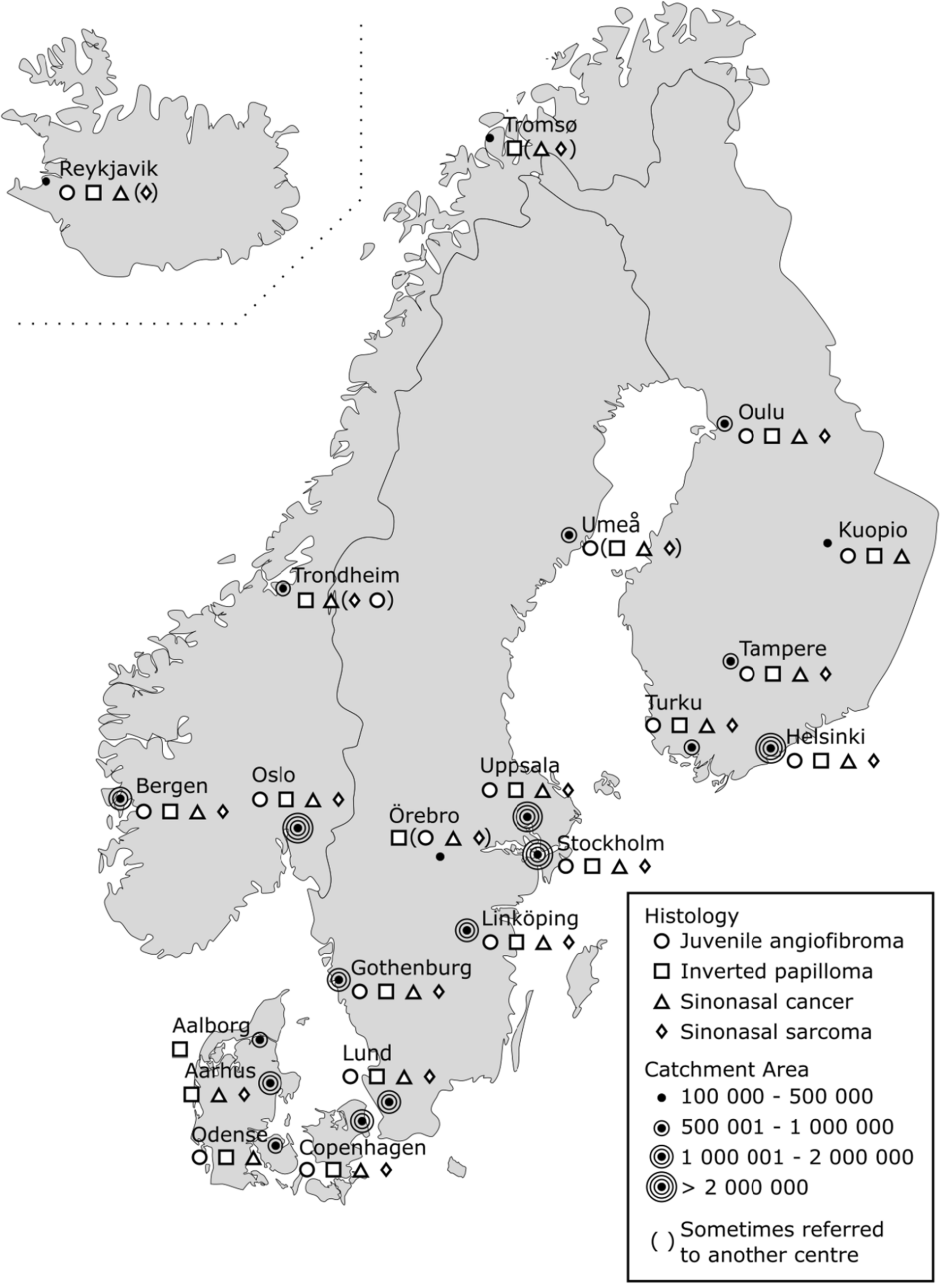
Catchment area and course of action according to tumour histology at the 21 Nordic university centres according to the respondent (Modified, source Wikimedia Commons/(CC-BY-SA-3.0)

Twenty (95%) out of 21 hospitals routinely used the endoscopic approach in the management of benign sinonasal tumours. Twenty centres (95%) treated sinonasal malignancies and 18 (86%) of them utilised the endoscopic approach routinely. Endoscopic surgery of benign tumours was centralised to certain surgeons at 17 (81%) centres and all 21 centres centralised the endoscopic surgery of malignant tumours. All centres had image-guided navigation available for surgery of sinonasal tumours. IP was reportedly managed endoscopically at all centres. Fourteen of the centres performed transsphenoidal surgery mainly as a collaboration between neurosurgery and ORL-HNS, and in 5 and 2 centres, it was mainly performed by neurosurgery or ORL-HNS alone, respectively. Responses by each centre are presented in Table 1. In Sweden, 2 (29%) university hospitals reported to refer certain patients to another centre, and the corresponding figures being 1 (20%) for Finland, 2 (50%) for Norway, 3 (75%) For Denmark, and 1 (100%) for Iceland. Care pathways of choice for JNA, IP, malignant sinonasal tumours, and sinonasal sarcoma at the centres are summarised in Fig. 1.

**Table 1:** Number of endoscopic surgeons at all 21 Nordic university clinics and each centre’s response on the matter of endoscopic surgery, of benign and malignant sinonasal tumours, and the centralisation of these procedures to certain surgeons

Country	City	No. of endoscopic surgeons	Endoscopic surgery of sinonasal tumours		Centralisation of endoscopic surgery certain surgeons		Speciality mainly performing transsphenoidal surgery
			Benign	Malignant	Benign	Malignant	
Sweden	Stockholm	3–4	Yes	Yes	Yes	Yes	ORL-HNS
	Uppsala	3–4	Yes	Yes	Yes	Yes	Collaboration
	Gothenburg	3–4	Yes	Yes	Yes	Yes	Collaboration
	Lund	3–4	Yes	No	Yes	Yes	Collaboration
	Linköping	1–2	Yes	Yes	Yes	Yes	Collaboration
	Örebro	1–2	Yes	Yes	Yes	Yes	Collaboration
	Umeå	1–2	Yes	No	Yes	Yes	Collaboration
Norway	Oslo	3–4	Yes	Yes	No	Yes	Collaboration
	Bergen	3–4	Yes	Yes	Yes	Yes	Neurosurgery
	Trondheim	3–4	Yes	Yes	Yes	Yes	Neurosurgery
	Tromsø	1–2	No	Yes	Yes	Yes	ORL-HNS
Iceland	Reykjavik	1–2	Yes	Yes	Yes	Yes	Collaboration
Finland	Helsinki	≥ 5	Yes	Yes	No	Yes	Collaboration
	Tampere	3–4	Yes	Yes	Yes	Yes	Neurosurgery
	Oulu	3–4	Yes	Yes	Yes	Yes	ORL-HNS
	Kuopio	3–4	Yes	Yes	Yes	Yes	Collaboration
	Turku	1–2	Yes	Yes	Yes	Yes	Collaboration
Denmark	Copenhagen	3–4	Yes	Yes	Yes	Yes	Collaboration
	Odense	3–4	Yes	Yes	Yes	Yes	Neurosurgery
	Aalborg	3–4	Yes	No	No	Yes	Neurosurgery
	Aarhus	1–2	Yes	Yes	No	Yes	Collaboration

Within Sweden, the median distance from one centre to the nearest university hospital was 172 km (range 68–579 km), with a median travel time of 2.08 h (range 0.85–6.6 h). The corresponding figures were 167 km (164–287 km) and 2.18 h (1.85–3.55 h) for Finland, 478 km (463–1134 km) and 7.17 h (6.35–17.07 h) for Norway, and 133 km (114–178 km) and 1.52 h (1.17–2.07 h) for Denmark.

### Case-based questions

**Fig. 2:**
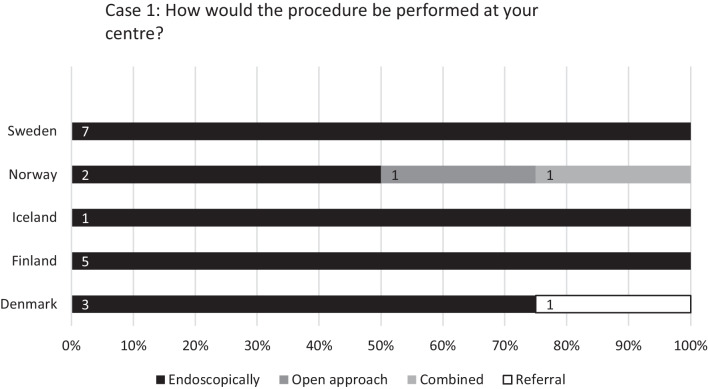
Responses to the question: “Case 1: A patient is to undergo medial maxillectomy for a small carcinoma limited to the area of the inferior turbinate and growing through the medial wall of maxillary sinus with no attachment to other walls of the sinus or the floor of the nose.” Percentages of university hospitals (Sweden 7, Norway 4, Iceland 1, Finland 5, and Denmark 4) on the *x*-axis and number of centres labelled on bars

The first case (see Fig. 2) described a patient with a small carcinoma of the inferior turbinate and the respondents were addressed regarding their method of choice for medial maxillectomy in similar cases. Eighteen (85.7%) of the centres chose endoscopic resection, 1 (4.8%) centre opted for open resection, 1 (4.8%) utilised a combined approach and 1 (4.8%) referred the patient to another centre.

**Fig. 3:**
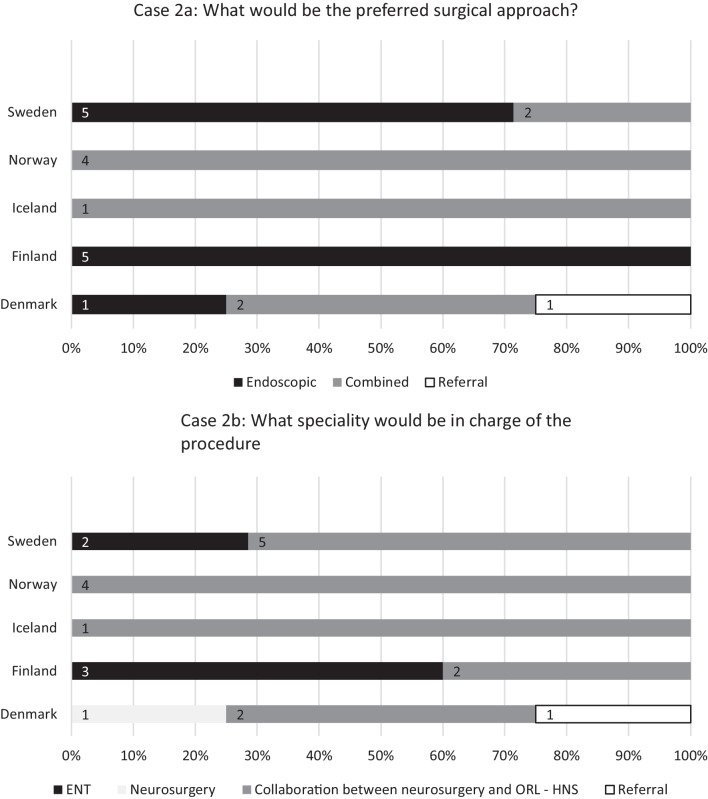
Responses to the question: “Case 2: A patient is diagnosed with esthesioneuroblastoma, Kadish C with involvement of the cribriform plate and the olfactory bulbs but no involvement of the brain.” Percentages of university centres (Sweden 7, Norway 4, Iceland 1, Finland 5, and Denmark 4) on the *x*-axis and number of centres labelled on bars. *ORL-HNS* otorhinolaryngology—head and neck surgery

The second case (see Fig. 3) described a patient with an esthesioneuroblastoma (Kadish C) with involvement of the cribriform plate and the olfactory bulbs, but no involvement of the brain. Eleven (52%) centres chose an endoscopic approach and 9 (43%) a combined approach. One (5%) centre referred the patient to another centre. The procedure was performed in collaboration between ORL-HNS and neurosurgery at 14 (67%) centres, by ORL-HNS or neurosurgery alone at 5 (24%) and 1 (5%) centre, respectively.

**Fig. 4:**
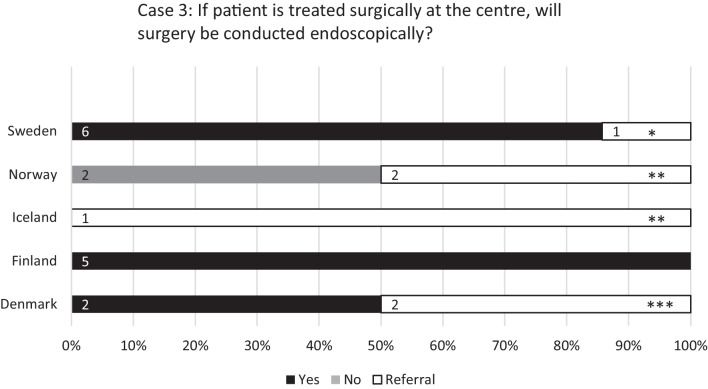
Responses to the question: “Case 3: A patient is diagnosed with juvenile angiofibroma of the sinonasal area with limited growth to the infratemporal fossa and limited to extracranial structures (f. rotundum not affected, classification Radkowski Ia-IIc, Fisch I-III)”. Percentages of university centres (Sweden 7, Norway 4, Iceland 1, Finland 5, and Denmark 4) on the *x*-axis and number of centres labelled on bars. *To Uppsala, **To Oslo, ***To Odense

The third case (see Fig. 4) described a patient diagnosed with JNA of the sinonasal area with limited growth to the infratemporal fossa and limited to extracranial structures. Similar patients were treated in-house at 15 (71%) of the centres. Resection was conducted endoscopically at 13 (62%) centres and through open approach at 2 (10%). Preoperative embolisation was preferred at all centres. Six (29%) centres reported referring similar cases to another hospital.

**Fig. 5:**
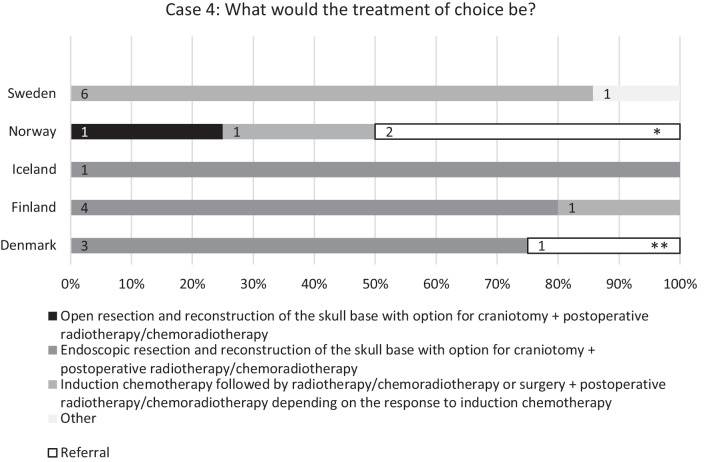
Responses to the question: “Case 4: Patient is diagnosed with SNUC (sinonasal undifferentiated carcinoma), with involvement of horizontal anterior skull base but no involvement of the dura (T4a). Thinning of the ipsilateral orbital medial wall but no involvement of periorbita or intraorbital growth. No involvement of the pterygoid plates or sphenoid/frontal bone. No metastases.” Percentages of university centres (Sweden 7, Norway 4, Iceland 1, Finland 5, and Denmark 4) on the *x*-axis and number of centres labelled on bars. *To Oslo, **To Aarhus

The fourth case (see Fig. 5) described a patient diagnosed with sinonasal undifferentiated carcinoma (SNUC), isolated to the horizontal anterior skull base with thinning of the ipsilateral orbital medial wall but no involvement of periorbita or intraorbital growth, no involvement of the pterygoid plates or sphenoid/frontal bone and metastases. Eight (38%) centres reported their treatment of choice to be endoscopic resection with the option for craniotomy, followed by reconstruction of the skull base and postoperative radiotherapy (RT) or chemoradiotherapy (CRT). Eight (38%) centres chose induction chemotherapy (ICT) followed by RT/CRT or surgery followed by RT/CRT, depending on the response to ICT. One (5%) centre chose the strategy of open resection and reconstruction of the skull base followed by postoperative RT/CRT and one (5%) centre chose RT/CRT depending on tumour stage. Three (14%) centres referred the patient to another centre.

## Discussion

We conducted a web-based survey to assess treatment practices of sinonasal tumours in the Nordic countries. Responses were received from all 21 Nordic university hospitals and thus the achieved results should accurately represent the current state of the treatment in this area. These responses indicate that the endoscopic approach is widely utilised, and most centres had several surgeons performing endoscopic tumour surgery. Thus, the prerequisites of effective endoscopic management of sinonasal tumours seem adequate. However, some variance in the utilisation of endoscopic surgery became apparent between countries, but also between centres within the same country.

The first case scenario in the questionnaire generated a nearly unanimous response regarding endoscopic resection as the method of choice for medial maxillectomy, although the centre reportedly opting for open approach reported to routinely utilise endoscopic resection in cases of malignant tumours. Furthermore, the Norwegian guidelines [9] state endoscopy as an alternative for medial maxillectomy. So does the position paper published in 2010 by the European Rhinological Society and the national guidelines of the United Kingdom published 2016 [1, 2].

When considering the case describing an esthesioneuroblastoma, Finland, Norway and Iceland exhibited national consensuses regarding the choice of surgical approach, while in Sweden and Denmark, both endoscopic and combined approaches were utilised. As no centres reported to use the open approach alone, treatment protocol seems in line with the contemporary literature on esthesioneuroblastoma. In their 2019 International Consensus Statement, Wang et al. [10] concluded that the endoscopic approach results in at least comparable survival outcome with the open resection of esthesioneuroblastoma, reporting reduced complication rates, reduced length of stay, improved quality of life, and reduced approach-related morbidity. Their study indicated that the endoscopic approach should be utilised for tumours classified as Kadish A-B and also for relatively local Kadish C tumours (as in the case described in the questionnaire). In more recent literature, both Spielman et al. [11] and Ngo et al. [12] reported the endoscopic and combined approaches, respectively, as oncologically comparable and less invasive for patients with esthesioneuroblastoma. Yet, there is some scarcity in the literature on comparisons between endoscopic and combined approaches. The variance observed regarding the speciality in charge of comparable cases is probably due to local tradition or administrative arrangements and is unlikely to impact the outcome of the treatment.

The case regarding JNA showed the highest rates of referral to another centre. This could be argued to mirror the rarity of the tumour, with a yearly incidence of 0.4/1,000,000 reported by Glad et al. in a Danish nationwide study [13], combined with the technical challenges JNA surgery pertains. Regarding surgical method of choice, the international Nordic consensus was reportedly endoscopic resection, with the exception of Norway, who reported not to utilise endoscopic resection. Whether the Norwegian centres opted for an open or combined resection, or a completely different approach, was not specified in the response. Preoperative embolisation of the tumour was unanimously advocated for among all centres and thus all centres operate in line with the recent literature [10, 14]. Regarding surgical approach, Wang et al. [10] concluded that the endoscopic approach resulted in at least comparable recurrence rates compared to the open approach and also reduced blood loss and morbidity, in cases of early stage tumours. They also reported at least comparable recurrence rates for the endoscopic approach regarding advanced tumours and concluded it to be a viable approach in select cases. In their systematic review of 75 studies with a total of 1586 patients, Jurlina et al. [15] reported comparable recurrence rates for both endoscopic and combined approaches. The open approach was associated with significantly higher recurrence rates.

When presented with the case of SNUC, the most popular management scheme was a tie between endoscopic resection and reconstruction followed by postoperative RT/CRT, and ICT followed by RT/CRT or surgery and postoperative RT/CRT depending on the response to the ICT. The centres in Denmark and Iceland unanimously opted for the former and so did most of them in Finland. The university centres in Sweden heavily favoured the route of ICT. The Norwegian consensus was split in two, divided between the route of ICT and open surgery followed by RT/CRT. The diversity in responses is somewhat expected, with the most advocated treatment being either definitive surgery followed by RT/CRT or ICT followed by RT/CRT with or without surgery [16–18]. Although in a recent review by Neo et al. [19], the conclusion was in favour of ICT, followed by definitive RT/CRT or surgery depending on the response, due to the benefits of chemoselection and possible advantages in distant metastasis rate. Regarding survival, Amit et al. [20] reported in favour of definitive CRT over definitive surgery in patients with favourable response to the ICT.

Responses to most presented clinical cases generated a somewhat varied response even nationally, despite national guidelines for each country with multiple university centres being available. One notable, recurring trend was the tendency to opt for an open approach at Norwegian centres. As no clear explanation for this was provided in the Norwegian national guidelines, it is to be assumed due to local tradition. The Nordic national guidelines available also exhibited varying degrees of comprehensiveness. For example, the Danish guidelines [21] recommended the use of the endoscopic approach and included a list of contraindications, the Swedish and Norwegian guidelines [9, 22] both listed the endoscopic approach as a viable option, but emphasised surgical experience and patient selection with no criteria stated, and the Finnish guidelines [23] did not consider surgical methods at all. With Hou et al. reporting only moderate general quality of head and neck cancer guidelines [24], it seems further research is needed for the creation of encompassing guidelines.

Denmark and Norway reported highest rates of referring patients to another centre. Regarding Denmark, this probably is due to the relatively short distances between the centres (Aarhus, Aalborg, Odense), making referrals logistically more feasible. According to responses from Norway, the smaller centres (Trondheim, Tromsø) opted to refer the patients to Oslo. This could mirror a more centralisation-oriented culture in Norway, with the distances being much greater than in Denmark and centres opting for Oslo over a more nearby centre. The Finnish Ministry of Social Affairs and Health issued a centralisation regulation [25] which stated that demanding surgeries performed less than 50 times a year in Finland should be nationally centralised and thus not performed at all five University hospitals. Considering the incidence for sinonasal malignancies in Finland, this regulation should apply to all sinonasal malignancies. Assuming the Danish incidence of JNA is generalisable across the Nordic countries, only 2 cases would be treated per year in Finland, thus clearly warranting centralisation. Yet, Finland exhibited the lowest rates of centralisation. With the exceptional rarity of both sinonasal sarcoma and JNA in mind, one could surely advocate for Finland to follow its neighbouring countries in the matter of centralisation. Although as distances between centres in the Nordic countries generally are quite long, it is logistically challenging for patients to undergo cancer treatment, not to mention the scheduling and financial difficulties travelling far to a centre of excellence pertains. This may be one reason for choosing to treat mostly locally in Finland. Yet, with Finland’s population and area paralleling Norway’s and the considerably shorter distances between university hospitals, prerequisites for successful centralisation seem to be there. Another reason may be the desire to conserve the versatility of the university hospitals. Teitelbaum et al. [26] reported that centralisation to higher volume centres (> 1.67 patients/year) seems to benefit survival, in a series of squamous cell carcinoma (SCC) and Flukes et al. [27] reported better outcome associated with both high-volume centres for most sinonasal and skull-base tumours. Goel et al. [28] described that, in a series of sinonasal SCC, delays between surgery and radiation treatment independently affects survival negatively, whereas delays between diagnosis to surgery did not impact survival. Thus, one could possibly argue for spending the extra time after diagnosis referring a patient to a national centre of excellence, though Murphy et al. [29] demonstrated that time to initiation of curative treatment independently increased mortality risk, in a large series of head and neck cancers. Trama et al. [30] reported that the fulfilment of quality criteria, based on expert consensus, was suboptimal in a study conducted in Ireland, Italy, Netherlands, and Slovenia. In a study conducted in Belgium, Verleye et al. [31] described how the fulfilment of their quality criteria generally was below their set target level. Even though these studies do not consider the Nordic countries, there seems to be some indication that quality of care of head and neck cancer generally tends to be variable in Europe. Thus, all measures to improve quality of care available should be employed.

Even with responses from all Nordic university centres and thus good prerequisites for comparison and detection of discrepancies between treatment practices, our survey is not without limitations. We sought a pragmatic approach; with questions, each clinician could easily answer without having to verify details possibly not readily available to them. Thus, more detailed, and less accessible factors, such as patient volumes and demographics were left uncovered, factors that possibly could explain some responses further. As all patient cases presented were fictional, their descriptions were kept as general as possible, to achieve the most generalisable response. Due to this, but also patient confidentiality, no imaging files could be provided. As imaging is essential for accurate assessment of nearly all sinonasal tumours, the lack thereof might influence the interpretation of the cases and thus the responses. Although, as all Nordic guideline documents are text based, we believe a respondent would be able to provide a generalisable response based on the description alone. With only one respondent per centre, there is also a possibility of personal expert opinion influencing the response, despite local and national guidelines and both the questionnaire and accompanying cover letter being worded to ask for the consensus at the centre. Contacts around the Nordic university centres assisted in identifying the representative respondents and respondents were asked to forward the questionnaire, if they felt someone else would be more qualified to respond accurately. As the questionnaire was web-based, there is always the, however, small, risk of miss-clicks. It is also worth noting that the percentages presented in Figs. 2, 3, 4, and 5 give the visual impression of larger discrepancies within countries with fewer university centres. Furthermore, it is recommended for the treatment to be individually tailored to each patient by a multidisciplinary tumour board [32, 33]. Thus, even minute details can alter the treatment plan and these general answers do not necessarily reflect all local factors that might influence it. There might also be some local variance in the execution of these meetings, e.g. which histologies are included to be discussed.

## Conclusions

The endoscopic approach for management of sinonasal tumours is utilised throughout the Nordic countries. Although there are national guidelines, no unified Nordic protocols for management of sinonasal tumours exist, despite the comparable size of population and healthcare systems in these countries. This becomes apparent in the received responses, exhibiting some discrepancies between countries, but also on a national level. Furthermore, the rate of centralisation remains relatively low, despite the rarity of these tumours and described benefits of centralisation. Distances between university hospitals do not seem to be a crucial factor determining centralisation habits. Centralisation to Nordic centres of excellence could be a way to increase surgical experience and thus overcome some of the obstacles the rareness of this disease entity poses.

### Supplementary Information

Below is the link to the electronic supplementary material.

Supplementary file1 (PDF 1239 KB)

## Data Availability

Not applicable, as little to no analysis of data is performed, all data gathered is presented in the article.
