# Supplementary material for: Endoscopic management of sinonasal tumours in the Nordic university hospitals: a survey
Source: Eur Arch Otorhinolaryngol. 2023 Sep 21;281(2):785–94. doi: 10.1007/s00405-023-08229-w (PMC10796644; doi:10.1007/s00405-023-08229-w)
Supplement: Supplementary file 1 — Supplementary file1 (PDF 1239 KB) [file 405_2023_8229_MOESM1_ESM.pdf]

Form is scheduled: publicity starts 28.2.2022 10.45 and ends 28.4.2023 23.59

Endoscopic management of sinonasal tumours in the Nordic countries - Survey

Information

Please, answer all questions in accordance with the treatment practice at your centre, unless instructed otherwise.

The form can be saved and continued at a later time by filling out your email at the bottom of the form.

Date of survey completion *

Name *

Speciality *

Institution *

Department *

Email *

Can we contact you for further information if

--Select--

needed? *

General questions

1. How many inhabitants does your referral area approximately encompass?

- [ ] a. <100 000
- [ ] b. 100 000 – 500 000
- [ ] c. 500 001 – 1 000 000
- [ ] d. 1 000 001 – 2 000 000
- [ ] e. > 2 000 000

2. How many surgeons operate sinonasal tumours endoscopically at your centre?

- [ ] 1-2
- [ ] 3-4
- [ ] 5 or more

3. Juvenile angiofibromas (choose the most suitable option)

- [ ] Are always managed at your centre
- [ ] Are sometimes managed at your centre
- [ ] Are always referred to another centre

If referred, where are patients referred to?

4. Inverted papillomas (choose the most suitable option)

- [ ] Are always managed at your centre
- [ ] Are sometimes managed at your centre
- [ ] Are always referred to another centre

If referred, where are patients referred to?

5. Malignant sinonasal tumours, excluding skin cancer (choose the most suitable option)

- [ ] Are always managed at your centre
- [ ] Are sometimes managed at your centre
- [ ] Are always referred to another centre

If referred, where are patients referred to?

6. Sinonasal sarcoma (choose the most suitable option)

- [ ] Are always managed at your centre
- [ ] Are sometimes managed at your centre
- [ ] Are always referred to another centre

If referred, where are patients referred to?

7. Is image-guided navigation available for surgery of sinonasal tumours?

- [ ] Yes
- [ ] No

Questions regarding endoscopic surgery

8. Is endoscopy routinely utilised in surgery of

8.1 Benign tumours?

- [ ] Yes
- [ ] No

8.2 Malignant tumours?

- [ ] Yes
- [ ] No

9. Is endoscopic surgery of sinonasal tumours centralized to certain surgeons?

9.1 Benign tumours?

- [ ] Yes
- [ ] No

9.2 Malignant tumours?

- [ ] Yes
- [ ] No

10. Which speciality mainly performs transsphenoidal surgery at ypur centre?

- [ ] ENT
- [ ] Neurosurgery
- [ ] Collaboration between ENT and neurosurgery

11. Are inverted papillomas mainly managed endoscopically?

- [ ] Yes
- [ ] No

Case-related questions

12. Case 1: A patient is to undergo medial maxillectomy for a small carcinoma limited to the area of the inferior turbinate and growing through the medial wall of maxillary sinus with no attachment to other walls of the sinus or the floor of the nose.

12.1 How would the procedure be performed at your centre?

- [ ] Endoscopically
- [ ] Open approach (e.g. lateral rhinotomy)
- [ ] Other

If other, please specify

13. Case 2: A patient is diagnosed with esthesioneuroblastoma, Kadish C with involvement of the cribriform plate and the olfactory bulbs but no involvement of the brain.

13.1 What would be the preferred surgical approach?

- [ ] Endoscopic
- [ ] Open
- [ ] Combined

13.2 What speciality would be in charge of this surgery at your centre?

- [ ] ENT
- [ ] Neurosurgery
- [ ] Collaboration between ENT and neurosurgery

14. Case 3: A patient is diagnosed with juvenile angiofibroma of the sinonasal areawith limited growth to the infratemporal fossa and limited to extracranial structures (f. rotundum not affected, classification Radkowski Ia-IIc, Fisch I-III)

14.1 Will the patient receive treatment at your centre or be referred to another centre?

- [ ] Treatment at your centre
- [ ] Referral

If referral, where? __________

14.2 If patient is treated surgically at the centre, will surgery be conducted endoscopically?

- [ ] Yes
- [ ] No

14.3 Will preoperative embolization be performed?

- [ ] Yes
- [ ] No

15. Case 4: Patient is diagnosed with SNUC (sinonasal undifferentiated carcinoma), with involvement of horizontal anterior skull base but no involvement of the dura (T4a). Thinning of the ipsilateral orbital medial wall but no involvement of periorbita or intraorbital growth. No involvement of the pterygoid plates or sphenoid/frontal bone. No metastases.

15.1 What would the treatment of choice be?

- [ ] Open resection and reconstruction of the skull base with option for craniotomy + post-op radiotherapy/chemoradiotherapy
- [ ] Endoscopic resection with option for craniotomy + reconstruction of the skull base + postoperative radiotherapy/chemoradiotherapy
- [ ]

Induction chemotherapy followed by radiotherapy/chemoradiotherapy or surgery+post-op radiotherapy/chemoradiotherapy depending on response to induction chemotherapy

- [ ] Other

If other, please specify

Comments

Comments or elaborations on questions above?

Continue, if needed

Partial submission

- [ ] I wish to save the form and continue using the link that will be sent into email I give

Email address

Proceed

Save

Prefilled form URL
